# CTCs 2020: Great Expectations or Unreasonable Dreams

**DOI:** 10.3390/cells8090989

**Published:** 2019-08-27

**Authors:** Elisabetta Rossi, Francesco Fabbri

**Affiliations:** 1 Department of Surgery, Oncology and Gastroenterology, Oncology Section, University of Padova, 35124 Padova, Italy; 2 Veneto Institute of Oncology IOV-IRCCS, 35128 Padua, Italy; 3 Istituto Scientifico Romagnolo per lo Studio e la Cura dei Tumori (IRST) IRCCS, 47014 Meldola, Italy

**Keywords:** CTC, heterogeneity, liquid biopsy, liquid surgery, clinical utility

## Abstract

Circulating tumor cells (CTCs) are cellular elements that can be scattered into the bloodstream from primary cancer, metastasis, and even from a disseminated tumor cell (DTC) reservoir. CTCs are “seeds”, able to give rise to new metastatic lesions. Since metastases are the cause of about 90% of cancer-related deaths, the significance of CTCs is unquestionable. However, two major issues have stalled their full clinical exploitation: rarity and heterogeneity. Therefore, their full clinical potential has only been predicted. Finding new ways of studying and using such tremendously rare and important events can open new areas of research in the field of cancer research, and could drastically improve tumor companion diagnostics, personalized treatment strategies, overall patients management, and reduce healthcare costs.

## 1. Introduction

In 2004, Cristofanilli and colleagues reported, for the first time, a trial regarding the level of circulating tumor cells (CTCs) in metastatic breast cancer. The trial results indicated this marker was a useful predictor of progression-free survival (PFS) and overall survival (OS) [1]. In 2007, the American Society of Clinical Oncology (ASCO) cited CTCs and disseminated tumor cells (DTCs) in recommendations on tumor markers. Ten years later, the American Joint Committee of Cancer (AJCC) proposed a new category for TNM staging in breast cancer M0(i+). The M0(i+) is defined as the presence of CTC or DTC, respectively, in the blood or in the bone marrow, in case of absence of clinical or radiological evidence of distant metastases. Fifteen years after the first Cristofanilli study, this marker was validated as a prognostic marker in a number of clinical settings, and few oncology topics have been so intensely investigated. [2,3,4,5,6]. However, due to their biological features, the technical hurdles regarding their investigation, and the lack of methodological standardization that make it difficult to compare different CTC studies [7,8,9,10,11], CTCs have still not reached the Olympus of the “clinical utility value”. Their predictive value, which we have been researching for decades, is still far from being fully demonstrated. Therefore, old promises should be carefully reconsidered and new directions should be undertaken. A first step in this direction was reported at the beginning of this year. Bidard and colleagues showed, for the first time, results on actual CTC clinical utility. They observed that CTC count, in ER+HER- metastatic breast cancer (MBC) patients, may be used to objectively choose the best therapy [12]. In addition, as other authors stated, another important challenge is how to trigger a paradigm shift in oncology research: anti-cancer personalized treatments should also strive to hit CTCs, and not only target the solid tumor compartment [13]. Recently, Gkountela and colleagues investigated CTC clusters. They observed that CTC clustering could direct the DNA methylation pattern. Specific FDA-approved drugs may take apart CTC clusters, triggering methylation remodeling and metastasis suppression [14]. This is a clear example of how it may be possible to bridge the gap between a potentially important marker, although still limited (i.e., the current CTC status), and a very useful theranostic approach, i.e., the new potential clinical utility of CTCs. Hence, although a lot of work has to be done, the aim is clear: Demonstrating and validating real CTC clinical utility, and looking for new CTC-targeting theranostic approaches.

## 2. Being Different, to Be Stronger

CTCs are a major cause of tumor relapse [15]. Disseminated into the bloodstream, CTCs are the “seeds”, able to give rise to new metastatic lesions. Hence, their significance is unquestionable. Despite the highly inefficient mechanism of spreading [16], some of them are able to survive into the bloodstream, resist biophysical and cell-mediated insults, and reach their final destination. There, they take root in the pre-metastatic niche, most probably already prepared and fueled by tumor cell-derived messengers. In the niche, CTCs lay dormant for an indefinite period, until some still little known signals trigger their lethal awakening [17,18,19]. CTCs are extremely rare and heterogeneous elements [20,21,22]. Although their quantification in the peripheral blood of cancer patients is quite unpredictable, even in patients with advanced disease, they have been found down to 1–10 cells/per ml of peripheral blood of metastatic breast, prostate, and colon cancer patients. Their number may be even lower in other cancers and/or in nonmetastatic setting [23,24,25,26]. Secondly, and equally important, CTCs are dynamically heterogeneous. Dynamic heterogeneity (DH) can be described as the characteristic of the tumor to evolve through space and time [20,27,28,29]. This event can produce solid masses composed of many clones, which may be different in transcriptomic, proteomic, and functional makeup. DH, and in particular that of the most aggressive tumors such as triple negative breast cancers (TNBCs), can be quite striking. It has been shown that no two single cells from TNBCs have an identical genomic profile [28]. CTC subpopulation onset and spreading may follow the solid tumor clonality and adaptability. Indeed, CTCs may acquire DH traits with time, from their onset, starting from a more epithelial-like phenotype and transitioning to a more mesenchymal-like state and vice versa, through processes known as epithelial-to-mesenchymal and mesenchymal-to-epithelial transitions (EMT and MET) [30,31,32,33,34,35]. In addition, EMT can be observed in distinct transitional states. These EMT states are fairly different and can be detected using cell surface markers and single-cell RNA sequencing. Indeed, EMT-hybrid CTC could have the highest metastatic potential with different degrees of aggressiveness due to their capabilities and proteomic and transcriptomic features [36,37]. These elements suggest that studying only a few biomarkers at a few time points, e.g., at first diagnosis and/or relapse, could only offer a very limited actionable vision of the disease. Monitoring the tumor evolution and progression through a timely and accurate multi-marker detection is a crucial investigation opportunity. CTCs lend themselves to this possibility, being a potential mirror/reflection of the evolving and progressing tumor. CTCs, indeed, are often genotypically, genomically, phenotypically, and functionally different. Some have stem cell features (e.g., CD44- or ABC-G2-positivity), others mesenchymal-like characteristics (e.g., N-Cadherin), others still, hybrids phenotypes [29,38,39,40]. With an appropriate technical set-up (Gallerani et al., unpublished), we could detect CTCs of all three types, at different sampling times, in the blood of esophageal cancer patients. Despite our current inability to see if other populations were present in the blood, our data agree with previously published reports and confirms that some CTC subgroups may be more dangerous than others may. Furthermore, some CTCs express endothelial markers and they reproduce vascular mimicry (VM), a phenomenon present in several human cancers associated with aggressive diseases. Williamson et al. demonstrated, in small cell lung cancer (SCLC), that these rare cells (VE-cadherin CTC) exhibit essentially the same copy number of gains and losses present in other CTCs and in ctDNA from the same patient, a highly related CNA profile and typical of SCLC [41]. We should also note that several CTC populations that coexist in the bloodstream could establish a relation with other normal blood cells. Recently, several authors demonstrated a crosstalk between different CTCs subpopulations and the immune system [42,43]. It is well-known that CTCs may express the receptor programmed death-ligand 1 (PD-L1) on their membrane [44]. PD-L1 is probably able to suppress the immune response against CTCs, helping their survival in the bloodstream [45,46]. Aceto and colleagues proposed a model where the association between CTCs and neutrophils supports the cell cycle progression during the blood trip [47]. The CTCs with the ability to survive in the bloodstream and to interact with leukocytes and platelets increase the possibility of forming metastasis. To this end, CTCs use several strategies to crosstalk with leukocytes and platelet:

(a) High expression of the immunosuppressive molecule PD-L1, which prevents T cell-mediated destruction [44,48,49,50];

(b) The expression of CD47, which provides a ‘don’t eat me’ signal [51,52,53];

(c) An altered expression of the apoptotic FAS and/or FASL proteins that may induce the apoptosis of T cells [54] or protect tumor cells from FAS-mediated apoptosis [55];

(d) The interaction with platelets, which induces EMT-like features in CTCs [56], and promotes tumor cell arrest and extravasation [57];

(e) Platelets promote the survival of CTCs during metastasis by conferring resistance to shear stress and attacks from NK cells [58,59];

(f) Platelet–CTC interaction can lead to the transfer of platelet MHC-I to tumor cells, thereby preventing the identification of NK cells and aiding the CTCs to escape from the cytolytic activity mediated by NK cells [60].

Furthermore, a CD14^+^CD11c^+^CD45^+^ myeloid subpopulation has been observed inside circulating tumor microemboli (CTM) or free in the bloodstream. Adams and colleagues named these cells ‘cancer-associated macrophage-like cells’ (CAMLs). Typically, these cells are giant (30–300 µm in length) with big multiple or polylobulated nuclei (14–64 µm in diameter) [61]. Adams et al. showed that CAMLs could be detected in the peripheral blood of patients with breast, prostate, pancreas, and lung cancer in percentages ranging from 81% to 97% of the total number of patients, contrary to healthy subjects, in which they are totally absent [61,62]. CAMLs also express epithelial markers, such as EpCAM and/or CK8/18/19. We still need to clarify if they directly express these epithelial markers (expression levels depend on the differentiation stage), or if they phagocytize material of epithelial origin. CAMLs seem to originate in the primary tumor and increase in blood samples from patients responding to radiotherapy, chemotherapy, or other treatments [63]. However, CAMLs have also been shown to actively interact with CTCs, or have a proangiogenic activity, as CD146 and TIE2 markers suggest [61]. In support of a pro-tumor role of these cells, in metastatic breast cancer patients, the presence of EpCAM+ CAMLs correlates with a shorter OS and PFS [64]. We could suppose that CAMLs might have an active role in helping CTC intravasation, extravasation, or survival in the bloodstream, thus participating in the metastatic process. The high degree of heterogeneity and complex relationships between CTC subpopulations and other blood “resident” cells, makes studying this biomarker more complex. It offers the opportunity to act both with drugs that affect CTCs, but also with drugs that limit the formation of clusters, as already mentioned [14], or with anticoagulant agents or those acting by stimulating the immune system to recognize CTCs [65,66].

Another pivotal aspect is the contribution of circulating cancer stem cells (C-CSCs), or stem-like CTCs. Cancer stem cells are cellular elements thought to propel cancer progression and to be responsible for the rooting of the disease in primary and metastatic sites, and hence, for local and distant recurrence. Although not fully determined, C-CSCs may derive from CSCs and have been identified in numerous types of cancer [40,67,68]. If CTCs can be seen as the overall population of tumor “seeds”, almost an epiphenomenon of cancer spreading, C-CSCs can be described as the actual first cause of metastasis, the proverbial needle in a haystack, since such events are even rarer than general CTCs. Furthermore, a close relation between EMT and C-CSCs has been shown, and an enhanced metastatic competence and a high drug resistance capability have also been described for these cells [69,70]. Despite being quite controversial elements, difficult to characterize specifically and unequivocally, C-CSCs may be considered key players in the genesis of cancer metastasis, and may clearly fulfill the model by Paget [16,71,72,73,74,75]. Specific C-CSC markers are difficult to find, but in general, some of them are fairly well established, e.g., CD133, CD44 (including different isoforms), ALDH1, and ABC transporters. A number of these markers have also been investigated in CTCs, also aimed at finding diagnostic, prognostic, and predictive indicators. None have been found, validated, and progressively translated into the clinical setting [25]. In 2017, Whang and colleagues showed that CTCs could act as potential precursor cells of metastasis and not only act in the intermediate step of metastatic cascade. Probably due to a stress response, CTCs could evolve in cells that were more aggressive and became tumor-initiating cells, probably different from the CSC-like cells present in the primary tumor [76]. The metastatic potential of EpCAM-positive CTCs has been confirmed using xenograft models in several papers [52,77,78]. A subset of CTCs showed a stem cell-like phenotype, these C-CSC have been detected in different kinds of cancer such as lung cancer with expression of BMI1, hepatocarcinoma where C-CSCs are CD90+CXCR4+, colon rectal cancer with CD44v9+ cells. In breast cancer one or more EMT markers, such as *TWIST*, *AKT2*, *PI3K*-*alpha*, *ALDH1*, were detectable on CTCs. This CTC subset highlights therapy resistance and a poor prognosis [68,79].

As already mentioned earlier, important papers have recently been published. Mirroring C-CSC potentialities, CTC-clusters have been demonstrated to be 23 to 50 times more capable of giving rise to actual metastases than single CTCs [80]. This capability has been explained by uncovering the link between the CTC cluster state and an increased accessibility of stemness-related transcription factors. CTC clustering is related to a DNA methylation pattern that promotes stemness and metastasis [14]. Again, the same group published a study on the striking importance of the cooperation between “normal” and cancer cells. The association of CTCs with neutrophils induces a proliferative boost, making them more competent in metastasis formation [47,81,82].

In conclusion, a number of elements may induce a CTC to gain a CSC-like cluster-state with specific cellular, molecular, and functional phenotypes and capabilities. More than ten years ago, the exposome was described as the overall environmental complement to the genome in determining the risk of a disease. It can be seen as the totality of exposures throughout one’s lifespan [83]. Hence, it can be inferred that in addition to genetic predisposition, every molecule or element that can prompt tumor onset and growth can be seen as exposome, or, more specifically, cancer exposome [Fabbri et al., under review]. Consequently, cancer exposome could include inflammation-related molecules, “normal” cellular elements associated to cancer progression (e.g., CAMs, CAFs, and platelets), environmental factors, and other molecules, i.e., elements known to be connected to tumor development, such as induction of EMT, C-CSCs, and progression. It becomes clear that the cancer exposome could contributes to the onset of CSC-like CTCs. Only a focused and multidisciplinary approach may truly help in finding innovative paths to unravel the enigmatic Gordian knot that may well represent cancer metastasis. In the light of recent results and these exposome-related concepts, we have to strive to find innovative treatments to suppress the spread of cancer.

## 3. Clinical Data: Hopes of Utility

In clinical practice, after pre- and analytical standardizations and diagnostic accuracy evaluations, a test has to demonstrate that it leads to health benefits [84] before being introduced in the decisional path.

Regarding CTCs, the multivariate analysis demonstrated that the CTCs count was the strongest prognostic biomarkers for patients survival [3,85]. For this reason, CTCs could be used to stratify the disease, but this biomarker is rarely used in clinical practice. Budd and colleagues studied 138 MBC patients, who underwent CT scans before and after start treatment. They compared the results with the CTC count obtained at baseline and after four weeks from the start of therapy, and demonstrated that CTC count is more reproducible than radiological response. This suggests that CTCs are a superior surrogate endpoint [86]. In metastatic breast cancer, the first trial designed to study the clinical utility of CTC count, SWOG S0500, was not able to find a survival difference for patients stratified using CTC count. This negative result was because the study suffered from a design fault and not a failure of CTC test [87,88]. In fact, cancers that show early resistance to first line chemotherapy are likely to be resistant to second line chemotherapy, whenever the second line is started. Moreover, it had already been reported that early changes of first line chemotherapy never demonstrated a gain in OS, whatever the technique used to guide the early change (functional imaging, etc.) [89]. Therefore, SWOG S0500 should not be regarded as the final proof that CTCs are not useful in patients’ management. However, because of these negative results, the 2015 American Society of Clinical Oncology, in its clinical practice guidelines, did not recommend the use of CTC count in women with metastatic BC [90]. In Europe, two other clinical utility trials based on CTC count are currently ongoing:

- The “CirCe01” trial (NCT01349842), similarly to SWOG0500, assesses early changes of CTC count during treatment in metastatic patients; patients were enrolled before the start of third line of chemotherapy (CT) and followed with the CTC test throughout the successive lines of CT.
- The “STIC CTC” trial (NCT01710605) investigated the clinical utility of the prognostic value of baseline CTC count. In this trial, patients were randomized in two arms: In the first arm, clinically driven patient’s treatment choose between CT and hormone therapy (HT) (CTC count not disclosed); in the second arm, CTC count driven treatment chooses patients. In fact, such as first-line treatment, patients with CTC ≥5/7.5 mL received CT, whereas patients with CTC <5/7.5 mL received HT.

As already discussed, this last trial demonstrated the clinical utility of CTC count in first line therapy in ER+HER2-MBC. Although results are awaiting a definitive publication, Bidard and colleagues have shown that in the majority of patients, the treatment chosen based on the CTC count was the same as the treatment chosen based on the clinic. On the contrary, in discrepant cases, CTC count may be more reliable for either escalating (i.e., considering CT in patients with high CTC count) or de-escalating (i.e., considering HT in patients with low CTC count) first line therapies [12]. SWOG0500 and CirCe01 have similar designs. Both studies evaluated CTCs at baseline and after treatment. In particular, CirCe01 evaluated CTCs number after every new line of CT in patients randomized in the CTC arm. The number of therapy lines represent the main difference between these two studies. In fact, CirCe01-enrolled patients about to start third line therapy (a population with a high chemo-resistance prevalence). On the contrary, SWOG500 enrolled patients just before first line CT. Moreover, CirCe01 trials, in nonrandomized run-in phase, also wanted to establish a CTC threshold to be used in the randomized part of the study. The STIC CTC, a randomized trial, focused on evaluating the health economic interest of taking into account CTCs to determine the kind of first line treatment for HR–positive MBC. Major limitations of this trial were a) the lack of standardized clinical criteria for recommending CT in the clinically driven arm, and b) that it was conducted prior to the widespread use of CDK4/6 inhibitors, now regarded as the standard of care for HR–positive MBC. The SWOG0500 was the first trial with a goal regarding the clinical utility of CTCs. However, the heterogeneity of first line treatments allowed in this trial might have variably influenced CTC behavior. Changing treatment to a different CT agent after the rise of early chemo-resistance may be not significantly effective, and this most probably influenced the results of the SWOG0500 [91]. A similar problem, given the design of the study, could have occurred in CirCE01. The STIC CTC trial had a different study design and, as preliminary results presented at the San Antonio Breast Cancer Symposium showed, it suggested that the CTC count could allow for identifying patients in whom the dose of therapy can be scaled. If the preliminary results of this study are confirmed, this could lead to a benefit for patients who will be able to see reduced side effects of the treatment and, in the long term, an economic saving for the national health systems.

Although still not incorporating recently proposed drugs, e.g., cyclin-dependent kinase 4/6 inhibitors, and indicating to escalate therapy, while it is usually suggested to try to de-escalate regimens in this clinical setting, the STIC CTC trial is the first investigation that showed an actual CTC-based clinical benefit. Hopefully, this huge step should be the first of a long series, prompting further investigations and highlighting the pivotal importance of clearly and specifically designed future clinical studies not to fall into previous misleading results.

Several studies evaluated the impact of CTC count and CTC characterization in monitoring treatment. Indeed, in metastatic castration-resistant prostate cancer (mCRPC), CTC elimination (CTC0) after short-term treatment proved to be an early response endpoint [92]. In this study, 3000 men were enrolled in five phase III trials. The response measures were evaluated regardless of the specific intervention and the changes in CTC status from CTC-positive (T baseline) to CTC-negative (after 13 weeks). This change was shown to be strongly associated with longer survival. Interestingly, the percentage change from baseline of prostate specific antigen (PSA), which is more widely used as end-point, did not discriminate survival at the same level of CTC0. In four out of five trials, the treatments included HT; HT can induce a PSA-level modulation, independent of an effect on cell killing. This HT-based effect limits the role of post-therapy PSA change measures as a reliable indicator of efficacy. In this study, CTCs allowed researchers to determine treatment response seven to eight weeks earlier than determined by standard RECIST criteria and PSA. For these reasons, CTC number, a biomarker that is not affected by modulations in androgen receptor signaling, should be included in clinical management of patients. It is important to note that this result could allow for the prevention of drug toxicity and cause a significant health savings [12,93,94,95].

## 4. Challenging the Current Paradigm: Liquid Surgery Premises and Hopes

So far, systemic therapies have aimed at preventing relapse after the removal of the primary tumor and delaying the already present metastatic tumor, confidently eliminating therapy-resistant clones and minimal residual disease. To contrast cancer dissemination, preventing metastasis in the first place, we think it is necessary to expand the conventional line of thinking that aims to hit an already widespread disease, and start new paths that anticipate its diffusion, e.g., directly and therapeutically targeting CTCs.

The investigation of the CTCs, and in general everything that can be included in the term liquid biopsy (LB), has aimed at simple, fast, and cost-effective monitoring of disease status or response to treatment. LB is less troublesome than tissue biopsy, thanks to the ease of access to body fluids, in particular when taking a tissue biopsy is often clinically impossible or not recommended [96]. Moreover, as already suggested, the tumor may change and tissue biopsies may not appropriately reflect the complex profile of a tumor disease, because of its dynamic heterogeneity (DH), which can only be addressed by taking biopsies from different tumor areas at different time points. Hence, LB may offer a more comprehensive cross-section of the disease.

LB can be a crucial alternative and/or addition to conventional and tissue-based diagnostic procedures. Targeting CTCs in the blood of patients could control or avoid metastasis spreading and the risk of relapse, just like the surgical removal of solid tumor can limit the disease or even cure a cancer patient. Therefore, two major lines of research could be followed to deplete CTCs as much as possible, striving not to leave any cells with actual metastasis-generating properties. One possible approach is to examine, in depth, CTC biology in order to identify actionable targets that could lead to their depletion and/or to make them harmless, as already cited [14,97]. A second possibility is to find new paths that can exploit already well-known aspects of CTCs: Their biological features, i.e., surface marker expression, dimension, clusterization potential, and their ecosystem, the blood flow.

Both ways are necessary to identify better-tailored CTC-based anticancer treatments for individual patients. The small number of CTCs per ml of peripheral blood is the major challenging physical limit. Although it may appear to be a simple solution, it is increasingly clear that it is necessary to extend the volumes of blood that should be analyzed and/or treated. Then, new approaches to overcome this obstacle, i.e., in vivo CTC collection and/or count devices, and apheresis-based procedures, are under investigation. The GILUPI CellCollector (CC) allows collecting CTC directly from the blood, in vivo. It is a functionalized medical wire covered with anti-EpCAM antibodies that, when inserted in the cubital vein of a patient for 30 min, could trap CTCs from up to one liter and a half of blood. Studies reported an increased number of CTCs detected compared to standard methods [98,99,100]. Recently, Didzdar and colleagues investigated the prognostic impact of CTCs rescued with CC in colorectal cancer (CRC) [101], but in contrast to other studies, they did not report prognostic significance of CC-CTC. The authors estimated that recovering CTCs with the CC is similar to extracting tumor cells from a blood volume range of 0.33–18 mL, a different volume compared to what was suggested in previous studies (volume range 1–3 L). Also, in our experience, the median number of CTCs retrieved from the blood of metastatic non-small lung cancer (NSCLC) patients was quite low (around three cells per CC device/patient (Fabbri et al., unpublished data). So, despite being a feasible [102] and promising idea, it seems that the CC has not maintained what it had predicted. In June 2019, Galanzha et al. exploited a photacoustic (PA) flow cytometry (PAFC) platform (cytophone platform) to count circulating melanoma cells in vivo. This method allows a noninvasive assessment of a large volume of blood using the PA effect as a transformation of absorbent laser pulse energy into sound through thermoelastic expansion of light objects. The probe is placed on the patients’ skin above the selected vein. The authors utilized 18 melanoma patients as the training set and another 10 new melanoma patients as the validation set. This system was able to detect circulating single melanoma cells, circulating clusters of melanoma cells, and it could discriminate between CMC cluster and circulating blood clots (CBCs). The PAFC detection limit achieved in vivo is about 1 CTC/1 L. The PACF-based CTC count needs further studies to confirm the great potential of this noninvasive method [103].

To avoid the bottleneck due to the low number of CTCs, the CTCTrap consortium and the Nick Stoecklein group used and validated the diagnostic leukapheresis (DLA) in order to enrich a higher number of CTCs. The CTCTrap efforts demonstrated that the procedure used allowed researchers to improve the number of CTCs isolated in MBC and metastatic prostate cancer (MPC), and that the procedure could be safely performed in different clinical sites [104]. The DLA product analyzed with Cell Search demonstrated a 30-fold increase in median CTC numbers detected [105] and an increase in the number of CTC-positive patients as well as M0 breast cancer patients [106]. This high number of enriched CTCs was exploited for the single cell molecular characterizations of CTCs [107] and also to culture CTCs [106]. Despite DLA enrichment, the CTC-cultured success is limited to few patients. Only in two out of eight patients were CTCs cultured from fresh and cryopreserved DLA [106]. The culture success rate was reached in samples with more than 300 CTCs, in agreement with other group results; this is probably because DLA samples with high CTC numbers and lower ratios of apoptotic CTCs were more likely to grow in culture. [106,108].

Usually, therapeutic apheresis is used for the rapid elimination of harmful or excessive blood components such as plasma proteins (plasma exchange) or for the harvest or elimination of cells (leukapheresis and platelet apheresis) and has found broad applications in a vast array of hematologic and onco-hematologic disorders. Keeping these reasoning in mind, removing CTC by filtration and adsorption, and reintroducing the blood without tumor cells back into the body would maximize CTC recovery from individual patients.

Statistically, it has been estimated that to find at least one CTC, the total blood volume (TBV) has to be analyzed, and that no less than 7.5 L of blood are necessary to detect at least 10 CTCs in almost every patient [109]. It is generally accepted that the TBV, i.e., the overall amount of fluid circulating within the arteries, capillaries, veins, and chambers of the heart, at any time in a typical adult human, is around 5 liters [110]. Interestingly, before metastasis onset, the presence of about 9 ± 6 CTC/L of blood has been mathematically predicted even in M0 breast cancer patients [111]. Scaling up this estimation, in low CTC number patients also, analyzing up to 72 L of blood would result in finding at least 200 to almost 1000 CTCs or more. Therefore, in order to treat a patient by eliminating up to all of the CTCs, it would be mandatory to perform an approach involving: a) A huge increase in the volume of blood to process, i.e., more than one TBV; b) a device that can catch all of the CTC subpopulations and that will not clog; and c) redirecting the blood into the patient after CTC removal. Although highly pioneering, we can predict that the removal of CTCs from the blood of a patient could control or even avoid metastasis spreading and the risk of relapse, just as surgical removal of a solid tumor can limit the disease or even cure a cancer patient. Such a kind of approach could be called liquid surgery (LS). A medical device able to eliminate CTCs from the blood circulation could substantially improve cancer management decreasing the overall disease burden, increasing OS and PFS, and could be used to support clinical decisions, as well as a higher DLA-based CTC retrieval [112,113]. In a long-term vision, it could even lower the need for high-cost therapeutic approaches and decrease therapy related toxicities. Preclinical tests and experimental set ups are currently running in our laboratories in order to improve and optimize such an approach. In our long-term vision, LB approaches will guide the decision of executing LS based on CTC presence in peripheral blood, exactly as a solid tumor is surgically removed after a standard tissue biopsy has revealed its presence and nature. LB could also monitor CTC levels after a LS procedure to evaluate depletion efficiency. Although reasonably invasive, the LS will be clinically feasible, not harmful, and manageable, comparable to a standard hemodialytic procedure used for kidney failure. The benefits of limiting, or even avoiding, metastasis onset have high preclinical and clinical potential [114,115] and will most probably exceed the possible drawbacks of extracorporeal circulation. Just like hemodialysis, a medical specialist will decide when the LS is needed, the therapeutic regimen, and the various parameters for the treatment. These would include frequency (e.g., treatments per week), duration of each treatment (e.g., up to 4 h or up to six months), and blood flow rates. As a maximum, up to three treatments per week, for up to 1–4 h per treatment, repeated during a period of up to six months, similar and in addition to an adjuvant therapeutic regimen, is predictable. Considering clinical parameters and patient conditions, the procedure could be repeated one or more times per patient per week, similarly to a standard chemotherapy infusion regimen. Of course, this pioneering and challenging idea has to be investigated in depth, tested, and validated, in vitro and in vivo, before becoming a reasonable treatment option that can be examined in a clinical setting. However, it remains a thrilling breakthrough hypothesis that should be put to the test.

## 5. Conclusions

In these last fifteen years, CTCs have been intensively studied in the field of cancer biology. CTC evaluation may provide clinically relevant and valuable information regarding cancer. The biological information gained from CTCs will be extremely important for opening new research fields, accurately targeting CTCs, and discovering new CTC-based treatment strategies. Multidisciplinary research approaches aimed at the overall “blood ecosystem”, an ecosystem for the CTCs and the metastatic cascade, are increasingly mandatory to fulfill this challenge. Monitoring CTC counts during therapy is a tool that may allow the assessment of disease development in real time, even prior to overt clinical signs of relapse. Targeting CTCs should become a translational objective that could pave the way towards increasing survival outcomes and reducing distal recurrence, preventing metastasis before it occurs. In the era of tailored therapy, precision oncology increases the range of treatment options, but to date, only a relatively small number of people could benefit. Moreover, to apply precision oncology is very expensive. The high cost is due to screening patients for tailored treatments and designing drugs that would be utilized in single patients or in limited groups of patients. We should not underestimate that CTCs could improve the health system costs and savings if the CTC-based diagnostic and therapeutic application could guide (a) better patient stratification, (b) first appropriate selection of personalized regimen, and (c) early treatment discontinuation and/or switch. The theranostic use of CTCs, which may be called LS, could establish a completely new area of research in the field of cancer research and management, and trigger the further improvement of CTC-based companion diagnostics.
